# Association fiber tracts related to Broca’s area: A comparative study based on diffusion spectrum imaging and fiber dissection

**DOI:** 10.3389/fnins.2022.978912

**Published:** 2022-11-07

**Authors:** Yupeng Wu, Jihui Liu, Guoning Yu, Ronghui Jv, Yibao Wang, Peizhuo Zang

**Affiliations:** ^1^Third Department of Neurosurgery, The People’s Hospital of China Medical University and the People’s Hospital of Liaoning Province, Shenyang, China; ^2^The People’s Hospital of China Medical University and the People’s Hospital of Liaoning Province, Shenyang, China; ^3^Department of Radiology, The People’s Hospital of China Medical University and the People’s Hospital of Liaoning Province, Shenyang, China; ^4^Department of Neurosurgery, The First Affiliated Hospital of China Medical University, Shenyang, China

**Keywords:** Broca’s area, diffusion spectrum imaging, fiber dissection, white matter, association fiber tract

## Abstract

Broca’s area, made up of Brodmann areas (BA) 44 and 45 in the ventrolateral frontal region, is associated with language production and articulation. A comprehensive network analysis of Broca’s area is necessary for understanding language function, which is still lacking. In this study, we attempted to investigate the association fiber tracts related to Broca’s area using both diffusion spectrum imaging (DSI) and postmortem fiber dissection. DSI was performed on 10 healthy subjects and an atlas comprising the average data of 842 healthy subjects from the Human Connectome Project. Fiber dissection was implemented in 10 cerebral hemispheres of cadaver donors. The following five association fiber tracts related to Broca’s area were identified: first, the distinct fasciculus of the inferior fronto-occipital fasciculus (IFOF), from Broca’s area (BA44, BA45) and pars orbitalis (BA47) to the parietal and occipital lobes; second, the ventral superior longitudinal fasciculus (SLF-III), from the supramarginal gyrus (BA40) to the ventral precentral gyrus (PreG, BA6) and posterior Broca’s area (BA44); third, the arcuate fascicle (AF), from the superior, middle, and inferior temporal gyrus (BA20, BA21, BA22) to Broca’s area (BA44, BA45) and ventral PreG; fourth, the frontal aslant tract (FAT), from Broca’s area (BA44, BA45) to the lateral superior frontal gyrus (SFG), medial SFG, and supplementary motor area (BA6, BA8, BA9); and fifth, the frontal longitudinal fasciculus (FLF), a novel intralobar frontal association fiber tract, from the anterior part of the middle frontal gyrus (MFG, BA46) and Broca’s area (BA45) to the caudal MFG (BA8), caudal SFG, and dorsal PreG (BA6). Moreover, compared with the left FAT, the right FAT covered almost the entire inferior frontal gyrus (BA44, BA45, BA47). The cross validation between DSI and fiber dissection revealed a good consistence in the association fiber tracts of Broca’s area. Combining DSI and fiber dissection, this study first identified five association fiber tracts related to Broca’s area and characterized their structure and anatomy comprehensively. The frameworks provided key elements for functional research in Broca’s area.

## Introduction

Language is a unique competency for humans that allows us to encode, elaborate, and communicate thoughts and experiences through words (Bernard et al., 2019). One of the great challenges is to identify candidate association fiber tracts involved in language production and processing. Broca’s area, consisting of the pars opercularis or Brodmann area 44 (BA44) and the pars triangularis or Brodmann area 45 (BA45), is associated with language production and articulation.

The classical Broca–Wernicke–Lichtheim model of language neurobiology has dominated the field for more than 100 years (Geschwind, 1970). This model, approved by numerous comparative architectonic studies and electrophysiological recordings, emphasized that Broca’s area was the center of language production. Recently, some functional neuroimaging studies have revealed Broca’s area also plays an important role in language processing (Price, 2010). However, significant gaps remain in understanding the structure–function overlap within Broca’s area. And it is unclear how Broca’s area communicates with other brain regions *via* association fiber tracts.

Although many efforts have been made, comprehensive delineation of fiber connections related to language processing is still a matter of debate (Leng et al., 2016; Panesar et al., 2017). Recently, diffusion tensor imaging (DTI), a fiber tracking technique of magnetic resonance imaging (MRI), has made it possible to display the fiber connections of Broca’s area *in vivo*. Nonetheless, there are still several limitations and pitfalls in the success of DTI fiber tracking, including the identification of false tracts and suboptimal coverage of small pathways or those with complex geometry (Thomas et al., 2014; Yeh et al., 2018). Moreover, there is a lack of comparative studies between gold-standard technique and DTI tractography. Historically, postmortem Klinger’s technique and neural circuit tracing are the best methods for validation (Campbell and Pike, 2014).

Diffusion spectrum imaging (DSI) involves a dense sampling of angular space for underlying water diffusion (Wedeen et al., 2008). By comparing DSI with radiographic techniques, Schmahmann et al. (2007) have shown that DSI has the potential to cast new light on the organization of the human brain in the normal state and in clinical disorders. Here, we employed DSI for acquisition reconstructed by GQI for estimation of fiber orientation as a high-angular-resolution-based approach (Fernandez-Miranda, 2013; Fernandez-Miranda et al., 2014). This approach leverages high directional sampling of diffusion imaging space to get a better resolution of the underlying white matter geometry for tractography (Wu et al., 2016b; Celtikci et al., 2018).

In this study, the comprehensive architectures of association fiber tracts in Broca’s area were presented and discussed, in order to show how language networks were organized. First, *in vivo* DSI was performed to characterize the association fiber tracts related to Broca’s area. Then, postmortem fiber dissection was used as a ground-truthing technique to validate DSI findings. Our study will provide an overall understanding of association fiber tracts related to Broca’s area, which have a great contribution to link fiber arrangements with function roles, e.g., language, in Broca’s area.

## Materials and methods

### Participants

High-resolution diffusion imaging data of 10 healthy subjects, who were right handed and 23–35 years old, were adopted as subject-specific deterministic fiber tractography from the Human Connectome Project (HCP) database.^1^ Meanwhile, the average high-resolution diffusion imaging data of 842 healthy subjects from the HCP were utilized to compile the average diffusion atlas.^2^ The HCP is a publicly available imaging database provided by the WU-Minn HCP Consortium (principal investigators: David van Essen and Kamil Ugurbil; 1U54MH091657) funded by the 16 NIH institutes and centers, as well as by the McDonnell Center (Van Essen et al., 2013). Moreover, 10 cerebral hemispheres without intracranial pathology were used to perform fiber dissection. All specimens donated to China Medical University were 35–63 years old and right handed. There were five left sides and five right sides and five males and two females.

This study was approved by the Ethics Committee of China Medical University (AF-SOP-07-1) and carried out in accordance with the Declaration of Helsinki. Written informed consents were obtained from the appropriate family member or substitute decision makers.

### Diffusion spectrum imaging tractography

#### Diffusion spectrum imaging data acquisition

Diffusion spectrum imaging data were acquired using a Siemens 3.0T Skyra system with a 32-channel head coil. A multi-shell diffusion scheme was used that had three diffusion gradient strengths, namely, 1000, 2000, and 3000 s/mm^2^. The *b*-value was sampled in approximately 90 directions. The in-plane resolution was 1.25 mm. The slice thickness was 1.25 mm (repetition time = 5500 ms, echo time = 89 ms, resolution = 1.25 mm × 1.25 mm, field of view = 210 mm × 180 mm, matrix = 144 × 168). This sequence was repeated two times for each slice.

#### Fiber tracking and reconstruction

Deterministic fiber tracking was performed using DSI Studio software (see text footnote 2). A stepwise approach was implemented to track fiber tracts as described previously (Wu et al., 2016a,b,c). The parameters were as follows: step size = 0.2 mm, minimum fiber length = 20 mm, turning angle threshold = 60°, and smoothing = 50%. The progressive voxels’ direction was approximately weighted by 20% of the incoming voxels’ direction and by 80% of its nearest fiber orientation. In the case of more than one fiber orientations in progression location, fiber orientation should be congruent with the incoming direction, and turning angle should be less than 60°. We pre-selected QA termination threshold by analyzing the number of false continuations generated within each subject’s dataset and chose the compromise value that allowed optimal anatomical detail with minimal noise. Fiber tracking was terminated in the following conditions: The quantitative anisotropy (QA) dropped below a subject-specific, pre-selected threshold value of between 0.02 and 0.08, 100,000 fiber tracts were generated, or fiber tract continuity no longer met the progression criteria (Yeh et al., 2013).

#### Fiber reconstruction

Diffusion data were reconstructed using generalized q-sampling imaging (GQI) with a diffusion sampling length ratio of 1.25 (Yeh et al., 2010). An automated anatomical labeling (AAL) atlas was used to parcellate the brain (Tzourio-Mazoyer et al., 2002). In order to generate the optimal fiber tract, we employed an AAL atlas-based approach in combination with a tractographic pattern of regions of interest (ROI) and regions of avoidance (ROA) (Fernandez-Miranda et al., 2014; Panesar et al., 2017). First, the AAL regions were warped to each subject’s diffusion matrix using the linear and non-linear registration algorithm feature in DSI Studio. This feature allowed the alignment of pre-defined cortical atlas regions to each subject’s unique diffusion map. This resulted in optimal atlas region placement without manual manipulation. Second, we used the SEED/ROI/ROA approach to obtain the exact fiber pathways in DSI Studio. For visualizing the connectivity profiles of each tract, we utilized the “endpoints-to-ROI” function in DSI Studio. This could convert fiber endpoints into larger cortical areas. Third, the endpoint ROI was also manually compared with superimposing specific AAL atlas regions. The tract origination/termination pattern was analyzed in this process. This process was done for each tract in both the subjects and the template, which guarantees the fiber tract was a true connection versus an artifact.

**FIGURE 1:**
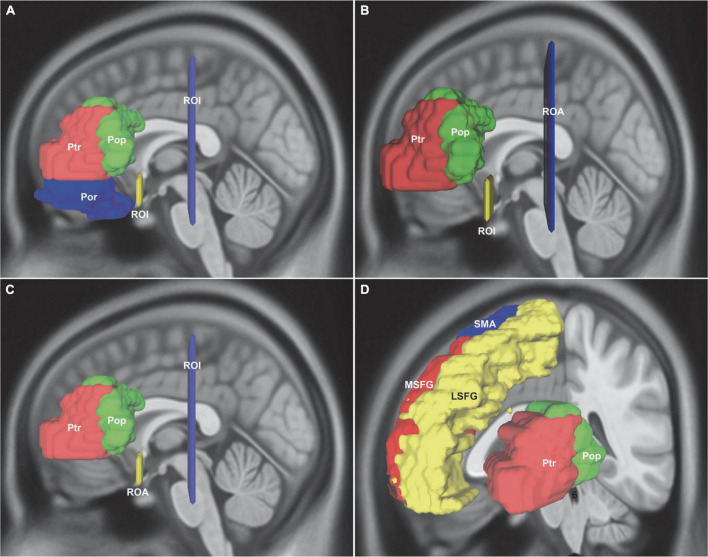
*In vivo* fiber tractography of the IFOF, UF, AF, SLF-III, and FAT around the Broca’s region (HCP-842). **(A)** Reconstruction of the IFOF using two ROIs: one located around the ventral part of the external capsule on the coronal QA color map and the other was on the coronal plane at the level of the central sulcus. **(B)** Reconstruction of the UF: the same ROI was used from IFOF and the other ROI was changed to the ROA. **(C)** Reconstruction of the AF and the SLF-III: the external capsule ROI was changed to ROA, whereas the central sulcus ROI was used. **(D)** For the FAT connection, the ROI seed regions included the LSFG, MSFG, SMA, Ptr, and Pop. IFOF, inferior fronto-occipital fasciculus; UF, uncinate fasciculus; AF, arcuate fascicle; SLF-III, superior longitudinal fasciculus III; FAT, frontal aslant tract; Pop, pars opercularis; Ptr, pars triangularis; Por, pars orbitalis; LSFG, lateral superior frontal gyrus; MSFG, medial superior frontal gyrus; SMA, supplementary motor area; ROI, regions of interest; ROA, region of avoidance.

For Broca’s area, the inferior frontal gyrus was parcellated into BA44, BA45, and BA47. First, for the inferior fronto-occipital fasciculus (IFOF), two ROI were drawn to select only the fibers that ran from the frontal region to the posterior region: One was around the ventral part of the external capsule on the coronal QA color map and the other was on the coronal plane at the level of the central sulcus (Figure 1A). Second, for the uncinate fasciculus (UF), the external capsule ROI was the same as that of IFOF. The other ROI was changed to region of avoidance (ROA) that prevented any posteriorly traveling fiber tracts (Figure 1B). Third, for the distinct fasciculus of the arcuate fascicle (AF) and the ventral superior longitudinal fasciculus (SLF-III), the external capsule ROI was changed to ROA. The central sulcus ROI was the same as that of IFOF (Figure 1C). Fourth, for the frontal aslant tract (FAT), the masks included the lateral superior frontal gyrus (LSFG), the medial superior frontal gyrus (MSFG), and the supplementary motor area (SMA) (Figure 1D).

#### Postmortem fiber dissection

In order to validate the DSI findings, postmortem fiber dissection was performed according to the technique described by Klingler (Dziedzic et al., 2021). Fiber dissection was undertaken at the Surgical Neuroanatomy Lab of China Medical University with the aid of microsurgical instrumentation and a surgical microscope (OPMI CS NC, 640 magnification, Carl Zeiss, Hohenstein/Breithardt, Germany). First, the specimen was fixed in 10% formalin for a minimum of 3 months. After rinsing with water for 2 days, the specimen was carefully peeled off the arachnoid and vessels and frozen at –15°C for 15 days. The freezing process could disrupt the gray matter, which could be removed more easily, and separate the white matter, which could facilitate the fiber dissection. Then, the specimen was thawed and fixed in 4% formalin solution for more than 5 days at room temperature. Before fiber dissection, the superficial anatomy of each cerebral hemisphere, including gyri and sulci, was studied carefully. The specimen was dissected in a stepwise manner from the lateral surface to the medial surface. The SLF-III and the AF were the first two association fiber tracts exposed after dissecting the surface of the frontal, occipital, parietal, and temporal cortices and the short U fiber tracts. The IFOF and the UF were the subsequently exposed fiber tracts after removing the insular cortex. The FAT and the crossing part of the FLF were the last exposed association fiber tracts. It was necessary to take photographs at each step as well as record the spatial relationships.

## Results

### Diffusion spectrum imaging findings

**FIGURE 2:**
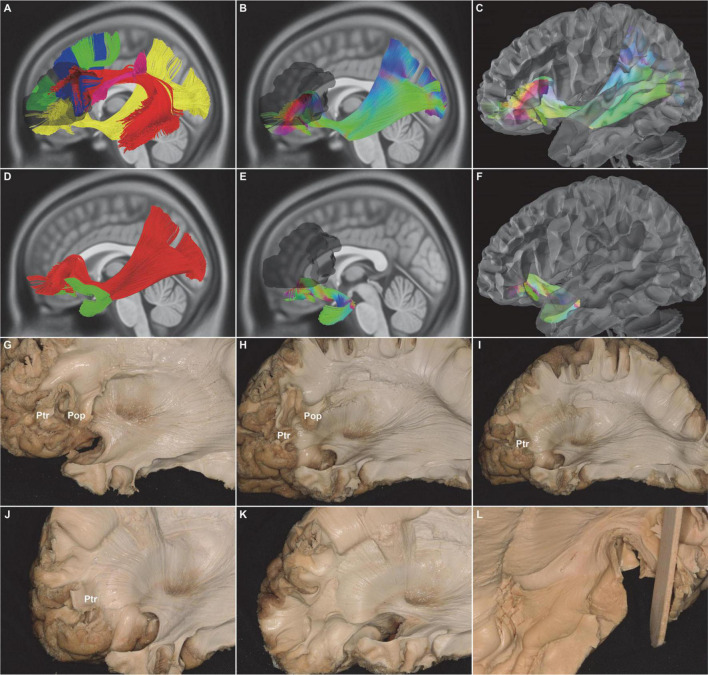
*In vivo* fiber tractography of the UF and the subdivision of the IFOF (HCP-842). **(A)** Five fiber tracts were identified around the Broca’s region (shadow area): the IFOF (yellow), AF (red), SLF-III (purple), FAT (blue), and FLF (green). **(B,C,G,H)** *In vivo* fiber tractography and fiber microdissection of the IFOF. The subdivision of the IFOF originated from the inferior frontal gyrus (i.e., Pop, Ptr, and Por), passed through in the depth of temporal lobe and insula, and finally projected to the occipital cortex, temporobasal areas, and superior parietal lobe. **(E,F,H,J, L)** *In vivo* fiber tractography and fiber microdissection of the UF. The UF connected the frontal and anterior temporal lobes as a “hook-shaped” fascicle. No fibers that connected with the Broca’s region were identified. **(D,H,I,J)** Spatial relationship of the IFOF and UF. IFOF was just above and medial to the UF at the level of the external/extreme capsule. **(G,H,I,J,K)** Step by step of the fiber microdissection of the IFOF frontal terminations. IFOF, inferior fronto-occipital fasciculus; UF, uncinate fasciculus; AF, arcuate fascicle; SLF-III, superior longitudinal fasciculus III; FAT, frontal aslant tract; FLF, frontal longitudinal fasciculi; IFG, inferior frontal gyrus; Pop, pars opercularis; Ptr, pars triangularis; Por, pars orbitalis; shadow area, Broca’s region.

In the DSI study, five association fiber tracts related to Broca’s area were identified, namely, the IFOF, the AF, the SLF-III, the FAT, and a novel fiber tract named as frontal longitudinal fasciculi (FLF) (Figure 2A). Both the anatomy and the spatial relationship of these tracts were described in the following.

The distinct fasciculus of the IFOF originated from the inferior frontal gyrus, including pars opercularis (BA44), pars triangularis (BA45), and pars orbitalis (BA47). Then, the fiber tracts narrowed into a bowtie shape at the limen insulae medial to the UF and subsequently extended through the anterior one-third of the superior limiting sulcus and the superior half of the anterior limiting sulcus. It eventually projected into the parietal and occipital lobes (Figure 2). Meanwhile, the UF, which makes up the second component of the indirect ventral pathway of the IFOF, was also tracked. The frontal terminations of the UF were at the inferolateral cortex of the frontal lobe, including the medial and lateral orbito-frontal areas (BA11), the pars orbitalis (BA47), the rectus gyri (BA11), and the minor branches of the middle frontal gyri (BA46) (Figure 2).

**FIGURE 3:**
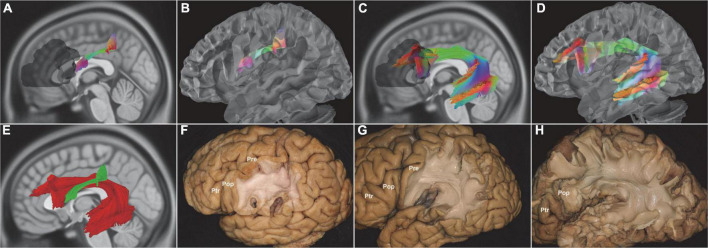
*In vivo* fiber tractography of the SLF-III and AF (HCP-842). **(A,B,F)** *In vivo* fiber tractography and fiber microdissection of the SLF-III. SLF-III connected the supramarginal gyrus with the ventral part of the precentral gyrus and Pop. **(C,D,H)** *In vivo* fiber tractography and fiber microdissection of the AF. The AF connected Broca’s area with the lateral temporal cortex *via* a dorsal projection arched around the Sylvian fissure. **(E,G)** Spatial relationship of the SLF-III and AF. The fibers of the SLF-III had a horizontal orientation laterally to the AF. AF, arcuate fascicle; SLF-III, superior longitudinal fasciculus III; Pop, pars opercularis; shadow area, Broca’s region.

The SLF-III was antero-posteriorly oriented fiber tracts that ran lateral to the AF. The SLF-III connected the supramarginal gyrus (BA40) with the ventral part of the precentral gyrus (PreG, BA6) and posterior Broca’s area [pars opercularis (BA44)] (Figure 3). The AF ran medial to the SLF-III. The AF connected Broca’s area with the lateral temporal cortex *via* a dorsal projection arched around the Sylvian fissure, which predominantly extended from the posterior superior temporal gyrus to the middle part of the superior temporal sulcus (BA22), the middle temporal gyrus (BA21), and the inferior temporal gyrus (BA20) (Figure 3).

**FIGURE 4:**
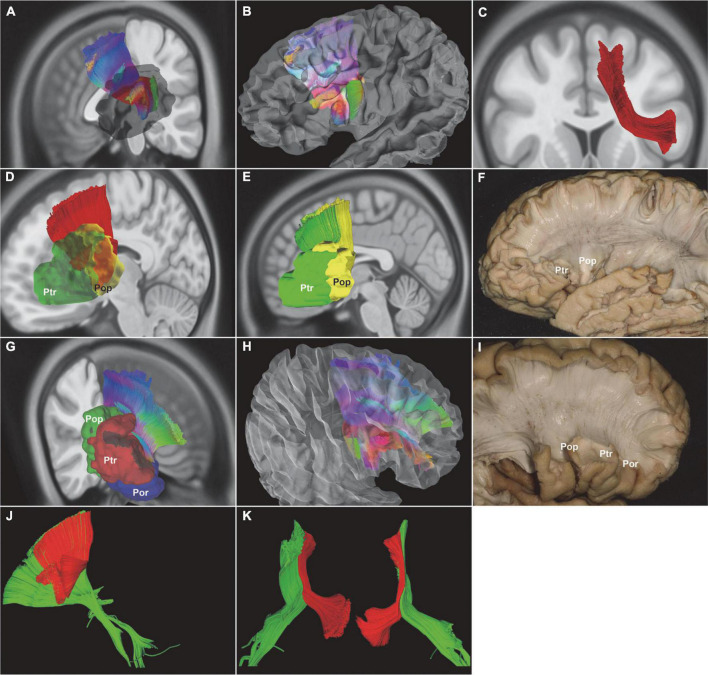
*In vivo* fiber tractography of the FAT (HCP-842). **(A–C,F)** The left FAT arose from the Broca’s region and projected to the SMA, MSFG, and LSFG. **(D,E)** Further analysis of the fiber contribution between the Ptr (green) and Pop (red). **(G–I)** The right FAT covered almost the entire length of the IFG. **(J,K)** The FAT fibers (red) were a very thin layer above the internal and external capsule fibers (green). FAT, frontal aslant tract; IFG, inferior frontal gyrus; LSFG, lateral superior frontal gyrus; MSFG, medial superior frontal gyrus; SMA, supplementary motor area; Pop, pars opercularis; Ptr, pars triangularis; shadow area, Broca’s region.

The left fiber tracts of FAT arose from the pars opercularis (BA44) and the pars triangularis (BA45), then sloped through the corpus callosum (CC), and projected into the SMA, MSFG, and LSFG (BA6, BA8, BA9). The most right fiber tracts of FAT arose from the pars opercularis (BA44) and the pars triangularis (BA45) and terminated in the almost SFG (BA6, BA8, BA9, BA10). A small right fiber’s tracts of FAT originated from the orbital cortex (BA47) and ended in the SFG (BA10) (Figure 4).

**FIGURE 5:**
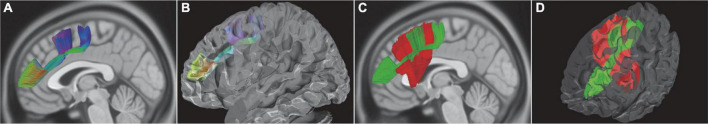
*In vivo* fiber tractography of the FLF (HCP-842). **(A,B)** The FLF ran as a longitudinal course approximately beneath and parallel to the MFG. Some bundles of fibers originated from the anterior part of the MFG and the other bundle of fibers from the Ptr and then ended in the caudal MFG, caudal SFG, and dorsal PreG. **(C,D)** Spatial relationship of the FLF and FAT. The FLF was intersecting together with the FAT. FLF, frontal longitudinal fasciculi; MFG, middle frontal gyrus; SFG, superior frontal gyrus; Ptr, pars triangularis; PreG, precentral gyrus.

A novel short intralobar fiber tract was identified and named as the frontal longitudinal fasciculus (FLF) according to its anatomical characteristics. The FLF ran through a longitudinal course. Some fiber tracts of FLF originated from the anterior part of the middle frontal gyrus (MFG, BA46) and other fiber tracts of FLF from the pars triangularis (BA45). Then, the FLF traced across the FAT inferior and medial to the MFG. The FLF ended in the caudal MFG (BA8), caudal SFG, and dorsal PreG (BA6) (Figure 5).

A subject-specific analysis revealed that the five association fiber tracts had similar location, shape, and trajectory in 10 subjects (Supplementary Figures 1–6).

### Fiber dissection findings

The SLF-III was the most superficial association fiber tract. There were several landmarks to locate the SLF-III, such as the supramarginal gyrus, the inferior frontal gyrus (IFG), the postcentral gyrus, the PreG, and the pars opercularis. The SLF-III started from the supramarginal gyrus (BA40) and terminated at the pars opercularis (BA44) (Figure 3). Then, the AF was deeply located medial to the SLF-III. The vertical segment of the AF started from the posterior temporal cortex. The inferior segment of the AF continued from the temporal regions to the frontal regions. Finally, the AF terminated at the pars opercularis (BA44) and the pars triangularis (BA45) (Figure 3).

Subsequently, it was the turn of the IFOF and the UF. They passed through in the deep brain, when the frontal, occipital, parietal, temporal, and insular lobes were moved away. The extreme capsule was the landmark to locate the IFOF and the UF. The IFOF started from the inferior frontal gyrus, just above the UF. It immediately became narrow and thin. Then, the IFOF passed backward within the temporal lobe and reached the occipital regions. Notably, the IFOF was clearly divided into superficial and deep parts at the ventral part of the external capsule. The superficial part of the IFOF arched medio-laterally at the level of the superior limiting sulcus of the insula and then terminated at the IFG (BA44, BA45, BA47). The UF turned inferiorly and reached the medial and lateral orbito-frontal cortices (BA11, BA47) (Figure 2).

For the FAT, the landmark was the SLF-III. In the opercular region, the two fiber tracts intersected with each other. The FAT was a quite thin oblique fiber tract, which was superficially adjacent to the fiber tracts of internal and external capsules. Thus, the microscope should be properly adjusted to guarantee the FAT continuity, especially the middle part, and avoid any confusion with the fiber tracts in the internal or external capsule (Figure 4).

**FIGURE 6:**
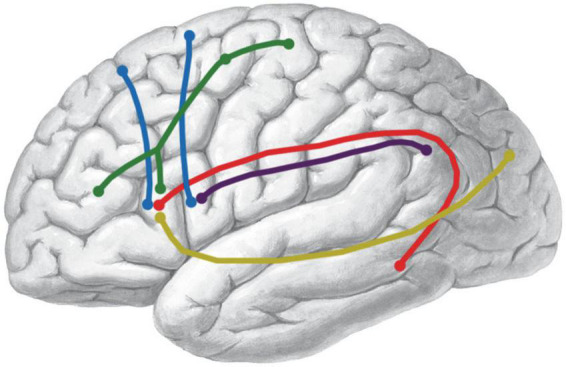
Schematic summary of the bundles connecting Broca’s area. The shape of the colored lines represents the rough anatomical path of each fiber tract. Color identifies different tracts. IFOF, yellow; AF, red; SLF-III, purple; FAT, blue; FLF, green.

The FLF and the FAT crossed each other to some extent. It was quite difficult to separate the FLF in the crossing regions. This could be due to the quality of the specimens and the limitation of the fiber dissection technique (Figure 6).

## Discussion

The language areas in the frontal cortex have been cytoarchitectonically defined by Broca in 1861 that span along a posterior-to-anterior gradient from the premotor cortex and BA6 in the PreG to BA44 and BA45 in the frontal cortex, which together constitute Broca’s area in the inferior frontal gyrus, and then anteriorly extended to the pars orbitalis (BA47) (Friederici, 2015). During speech perception, the association fiber tracts play a crucial role in fast transmission of electrical impulses between Broca’s area and other brain regions. Two main language processing streams connecting temporal and frontal areas were proposed: a ventral stream (the IFOF and the UF) supporting sound-to-meaning mapping and a dorsal stream (the SLF and the AF) subserving sound-to-motor mapping (Rauschecker and Scott, 2009). Recently, DTI has made it possible to display the fiber connections of Broca’s area *in vivo*. Although many efforts have been made, comprehensive delineation of fiber connections related to language processing is still a matter of debate (Leng et al., 2016; Panesar et al., 2017). Still, there is a lack of comparative studies between gold-standard technique and DTI tractography. Historically, postmortem Klinger’s technique and neural circuit tracing are the best methods for the validation (Campbell and Pike, 2014).

In order to solve the extant problems, this study first integrated DSI, a novel technique with high-spatial and high-angular resolution, with advanced reconstruction and deterministic tractography, which could map fiber connections much better than conventional DTI (Wang et al., 2013; Wu et al., 2016c; Yoshino et al., 2016; Panesar et al., 2019). Our study further applied anatomical fiber dissection, a gold-standard method, to validate the DSI findings. Our results revealed good concordance between DSI and fiber dissection and provided direct anatomical evidence for fiber connections in Broca’s area. Notably, the association fiber tracts related to Broca’s area was a solid confirmation by the subject-specific deterministic DSI tractography, the average atlas DSI tractography, and the 10 hemisphere fiber dissection, which not only showed individual differences of fiber connections, but also provided the average fiber connection patterns, as well as real-world fiber connections in Broca’s area.

### The inferior fronto-occipital fasciculus and the uncinate fasciculus

The IFOF and the UF are parts of the fronto-capsular white matter pathways, which participate in the formation of ventral language circuitry (Von Der Heide et al., 2013; Panesar et al., 2017). Previously, postmortem fiber dissection and DSI tractography have reported that the main course of the IFOF and the UF was at the level of the insula and the cortical terminations (Ebeling and von Cramon, 1992; Hau et al., 2016; Vassal et al., 2018). But the precise terminations in frontal cortex remain a major uncertainty. Kier et al. (2004) have noted that it is difficult to differentiate the two fiber tracts due to their frontal intermingling. Our study adopted DSI tractography to address the issue of crossing fiber tracts effectively (Fernandez-Miranda, 2013; Fernandez-Miranda et al., 2014).

The UF belonged to the limbic system, and connections with the amygdala, hippocampus, anterior temporal convexity, temporal poles, frontal poles, frontal basal area, and inferior frontal gyrus (Broca’s area) were used to be reported (Martino and De Lucas, 2014; Leng et al., 2016). In order to determine the cortical terminations of the UF and minimize *a priori* on the terminations, our study located the UF stem as the ROI and set the ROA posteriorly (Figure 1). This approach could ensure that all fibers passing through the UF stem were traced, and any IFOF fiber was excluded. Based on the priori anatomical knowledge and the “endpoints-to-ROI” function in DSI Studio software, we confirmed that the frontal terminations of the UF were mainly in the medial and lateral orbito-frontal areas (BA11), the pars orbitalis (BA47), the rectus gyri (BA11), and the minor branches in the middle frontal gyri (BA46). These were also verified by our postmortem fiber dissection findings. We also identified that there was no connectivity between the posteriorly cortical terminations and Broca’s area, amygdala, hippocampus, and parahippocampal gyrus.

In this study, the distinct fasciculus of the IFOF originated from the inferior frontal gyrus (BA44, BA45, and BA47), then formed a single stem which traversed the external capsule in the medial side of the UF, and finally projected into the occipital cortex. As the IFOF has multiple cortical terminations, some reports have suggested that the IFOF was a “multifunction” fiber tract with multi-component bundles (Panesar et al., 2017). Martino et al. (2010) have first proposed that the IFOF, only the posterior component, was a bilayer fiber tract, which arranged as superficial and deep parts in fiber dissection. Subsequently, Sarubbo et al. (2013) have investigated the frontal component of the IFOF using both fiber dissection and DTI tractography in a single left hemisphere. Our previous fiber tracking study has successfully identified five subcomponents of the IFOF (Wu et al., 2016b). In this study, the description of the IFOF conformed to the “IFOF-III” subcomponent.

### The arcuate fascicle and the superior longitudinal fasciculus

The SLF is one of the most voluminous fiber tracts, which has been well described previously. There remains some controversy concerning the SLF segmentation (Makris et al., 2005; Martino and De Lucas, 2014). Here, the widely accepted SLF segmentation was adopted for further study (Makris et al., 2005).

Conventionally, as the longest component of the SLF, the AF (SLF-IV) connected the lateral temporal cortex with the frontal cortex *via* a projection that arched around the Sylvian fissure. In early 1822, Takemura et al. (2020) proposed the existence of AF and described the detailed structure of AF. According to the previous studies, we noted that there were significant differences regarding the cortical connections of the AF. A recent review, with the focus on the cortical terminations of the AF, has shown that the AF ran from both the ventral part of the PreG and the posterior part of the pars opercularis to the temporal gyrus (accounting for 63.9%, articles number = 478). We considered that individual differences might contribute to the controversy of the cortical terminations. The limitations of fiber dissection and DTI tractography might be other reasons affecting the qualitative analysis of the AF connectivity. In this study, we adopted DSI tractography to reduce the problems of intersecting fiber tracts. Previously, the Klingler’s fiber dissection removed the extensive gray matter, even the adjacent white matter, which led to extensive losing of cortical landmarks. In attempting to solve these problems, DSI tractography was performed in prior to fiber dissection. Based on the “map” identified by DSI tractography, we could separate the target fiber tract carefully and accurately. Thus, the cortical terminations of a specific fiber tract could be reserved in maximum. Meanwhile, the anatomical knowledge and hands-on experience also played an important role.

Up to date, there is still a lack of evidence that the SLF-III played a role in language function. With the combination of fiber dissection and DSI tractography, our findings revealed that the AF and the SLF-III had similar fiber connectivity with the inferior frontal gyrus (BA44) and ventral PreG (BA6). According to the structural feature, we suggested that the SLF-III played a crucial role in the language circuitry.

### The frontal aslant tract and the FLF

As the third major fiber tract contributed to the speech network, the FAT has become a research hotspot (Chang et al., 2015). Catani et al. (2012) have first identified the existence of the FAT in the human brain, which connected the pars opercularis (BA44) with the pre-SMA mainly, and named it based on the structural feature of its aslant pathway. Afterward, there were few studies to validate the existence of the FAT in postmortem dissection. In this study, our findings have shown that the FAT closely intersected with the terminations of other association fiber tracts, especially the IFOF and the AF, at the level of the frontal operculum. It should be noted that artifacts and false bundles could occur by forcing the dissection in an orientation, but not following the natural axonal planes (Silva and Andrade, 2016). It could also lead to the failure of separating the FAT from the SLF-III and the AF following the standard procedures of fiber dissection (Bozkurt et al., 2016). The FAT was just a thin layer of fiber tract, evidently in the coronal view. The upper and middle parts of the FAT were adjacent and superficial to the cortico-spinal projections, fronto-thalamic projections, and fronto-striatal projections at the level of the CC. It was quite difficult to dissect the whole FAT fiber tract, which was fragile to break down. Thus, we speculated that there were some intertwines between the superior parts of the FAT and the internal or external capsule projections in most studies. In our experience, skilled fiber dissection, professional neuroanatomy knowledge, and fresh specimens were essential to obtain well-dissected FAT and informative fiber structure.

So far, little is known about the functional role of the FAT. In consideration of the FAT connectivity, most reports suggested that the left FAT played an important role in language processing. Catani et al. (2013) have first shown that the degeneration of the left FAT underlay the verbal fluency deficits, which has been widely accepted. In this study, our findings have shown that the fiber volume and the endpoints were significantly different between the left subdivisions of the FAT-Op and the FAT-Tr. Previous studies have comparatively analyzed the difference between the FAT-Op and FAT-Tr, as well as between the SMA and pre-SMA (Nachev et al., 2008; Kelly et al., 2010). Contrary to left lateralization of the FAT reported by previous studies, our tractographic findings have first demonstrated that it was only in the right hemisphere that the FAT connected the pars orbitalis (BA47) to the SFG (Szmuda et al., 2017; Pinson et al., 2022), which was consistent with the findings in the monkey brain (Yeterian et al., 2012). Thus, further functional studies should be paid more attention to the right FAT.

There were some studies that have given a comprehensive account of the short frontal lobe connections in monkeys (Yeterian et al., 2012). But, in view of cross-species differences, the reference value was limited in these reports. In our study, the anatomical findings of the FLF suggest that it is worthy to take more attention to the connectivity of the longer intralobar fiber tracts to distant cortical areas, such as the FLF connected to Broca’s area. Our study provides valuable evidence on the anatomy of the FLF, from morphology to spatial relations, which could facilitate understanding of the role of the FLF in language function.

### Limitations

There were several limitations in our study. First, although Klingler’s fiber dissection is widely considered as a useful tool for validation of fiber tractography, it is still susceptible to various limitations. It is particularly difficult in the dissection of the regions with different fiber terminations’ overlapping. This requires a good knowledge of the human white matter anatomy and a specific expertise. Second, a DSI template of 842 subjects is enough to analyze the average connectivity pattern of Broca’s area. However, the sample size of subject-specific healthy control was relatively small. For better understanding of the variability of the language networks in the human brain, a larger number of subjects will be needed with a balanced number of genders, ages, language skills, etc. Third, our study is merely a structural connectivity investigation. The potential functional roles of these five fiber tracts remain to be proven.

## Conclusion

In summary, to visualize the language networks of Broca’s area, this study integrated postmortem fiber dissections and *in vivo* DSI tractography and identified four major association fiber tracts, namely, IFOF, AF, SLF-III, and FAT. The exact cortical sites and comprehensive spatial relationship of the fibers, as well as the extensive and detailed anatomical descriptions of these fibers, were characterized. Moreover, for the first time, we reported a novel white matter tract, the FLF, that was confirmed in 10 subjects and a DSI template of 842 subjects. Compared with the classical Broca–Wernicke–Lichtheim model of language processing, the advanced neuroimaging applied in our study provided a more detailed description of the association fiber tracts related to Broca’s area. Our findings provided a solid theoretical foundation for the future functional studies.

## Data availability statement

The datasets presented in this study can be found in online repositories. The names of the repository/repositories and accession number(s) can be found in the article/Supplementary material.

## Ethics statement

This study was approved by the Ethics Committee of China Medical University (AF-SOP-07-1). The patients/participants provided their written informed consent to participate in this study.

## Author contributions

YiW and YuW performed the experiments. GY and RJ contributed to the data interpretation and picture preparation. JL contributed to the reagents, materials, and analyses tools. YuW and PZ wrote the manuscript and revised the manuscript. All authors contributed to the article and approved the submitted version.

## Footnotes

1
https://humanconnectome.org

2
http://dsi-studio.labsolver.org

## Supplementary material

Supplementary Figure 1 *In vivo* fiber tractography of the AF in all 10 subjects.

Supplementary Figure 2 *In vivo* fiber tractography of the FAT in all 10 subjects.

Supplementary Figure 3 *In vivo* fiber tractography of the FLF in all 10 subjects.

Supplementary Figure 4 *In vivo* fiber tractography of the IFOF in all 10 subjects.

Supplementary Figure 5 *In vivo* fiber tractography of the SLF-III in all 10 subjects.

Supplementary Figure 6 *In vivo* fiber tractography of the UF in all 10 subjects.

## Abbreviations

BA: Brodmann area
DSI: diffusion spectrum imaging
DTI: diffusion tensor imaging
QA: quantitative anisotropy
GQI: generalized q-sampling imaging
HDFT: high definition fiber tracking
ROI: region of interest
ROA: region of avoidance
IFOF: inferior fronto-occipital fasciculus
UF: uncinate fasciculus
SLF: superior longitudinal fasciculus
AF: arcuate fascicle
FAT: frontal aslant tract
FLF: frontal longitudinal fasciculi.
