# Supplementary figures and images for: Association fiber tracts related to Broca’s area: A comparative study based on diffusion spectrum imaging and fiber dissection
Source: Front Neurosci. 2022 Nov 7;16:978912. doi: 10.3389/fnins.2022.978912 (PMC9676966; doi:10.3389/fnins.2022.978912)

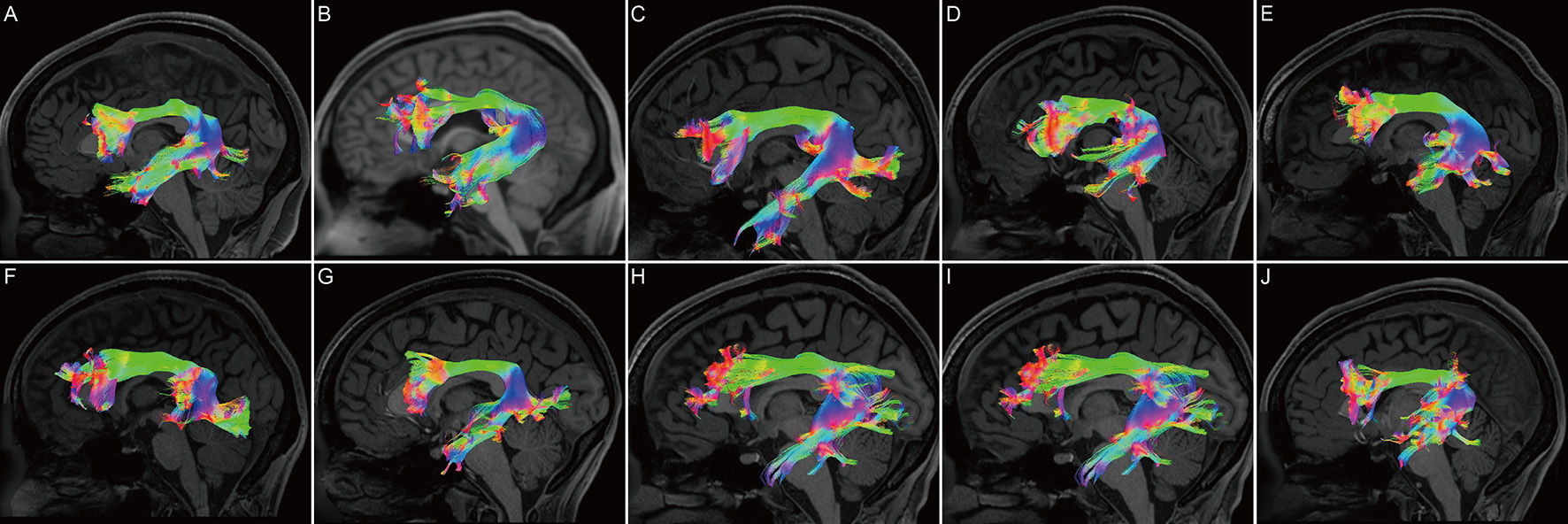

Supplement: Supplementary Figure 1 — In vivo fiber tractography of the AF in all 10 subjects. [file Image_1.JPEG]

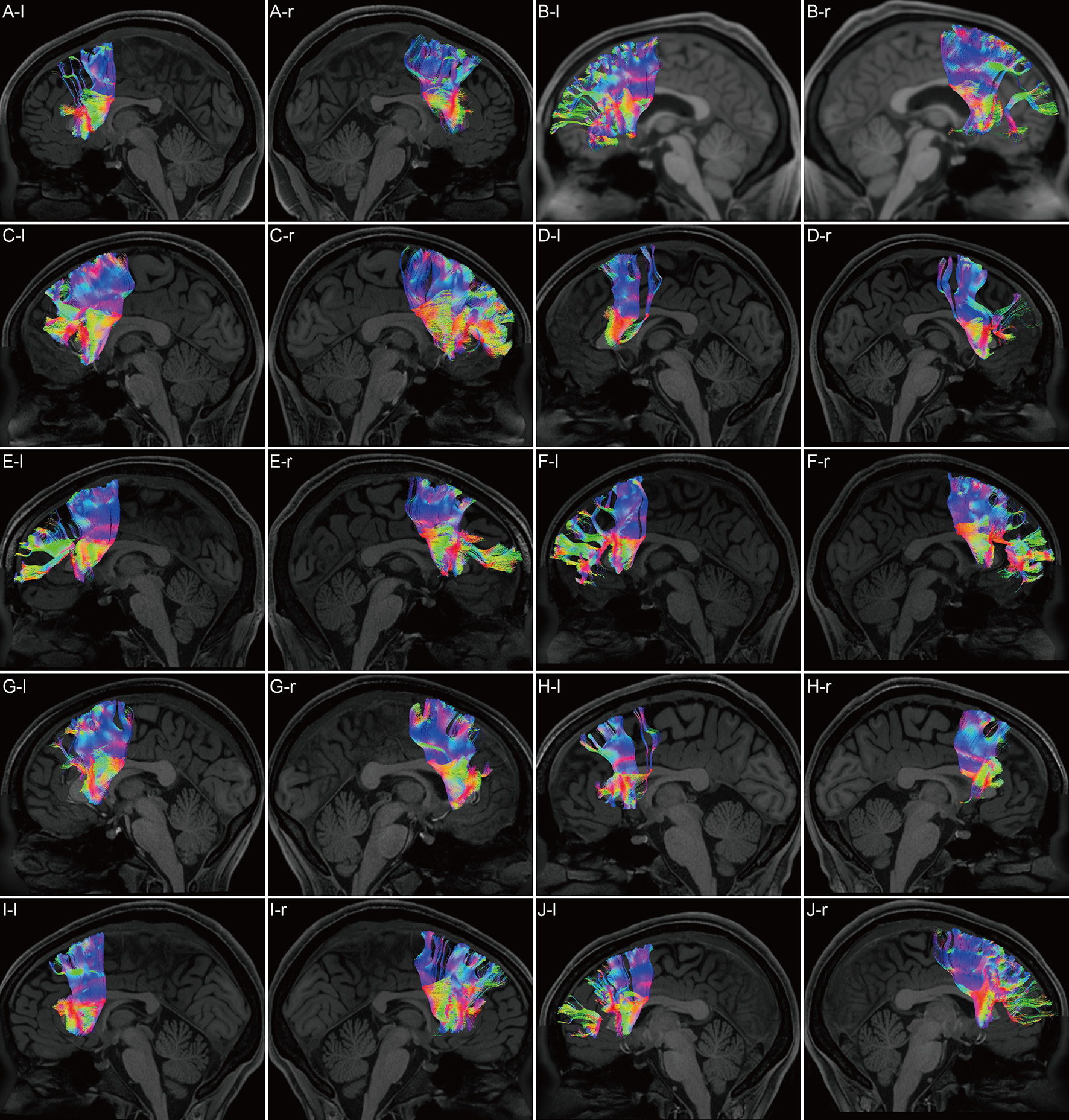

Supplement: Supplementary Figure 2 — In vivo fiber tractography of the FAT in all 10 subjects. [file Image_2.JPEG]

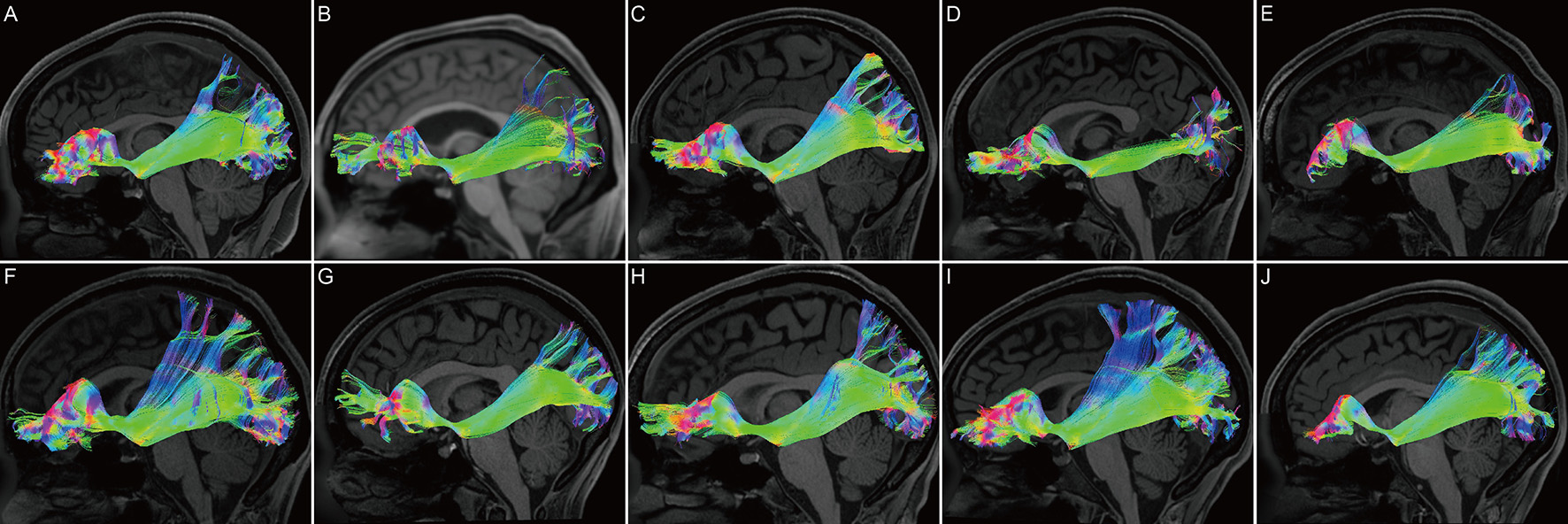

Supplement: Supplementary Figure 3 — In vivo fiber tractography of the FLF in all 10 subjects. [file Image_3.JPEG]

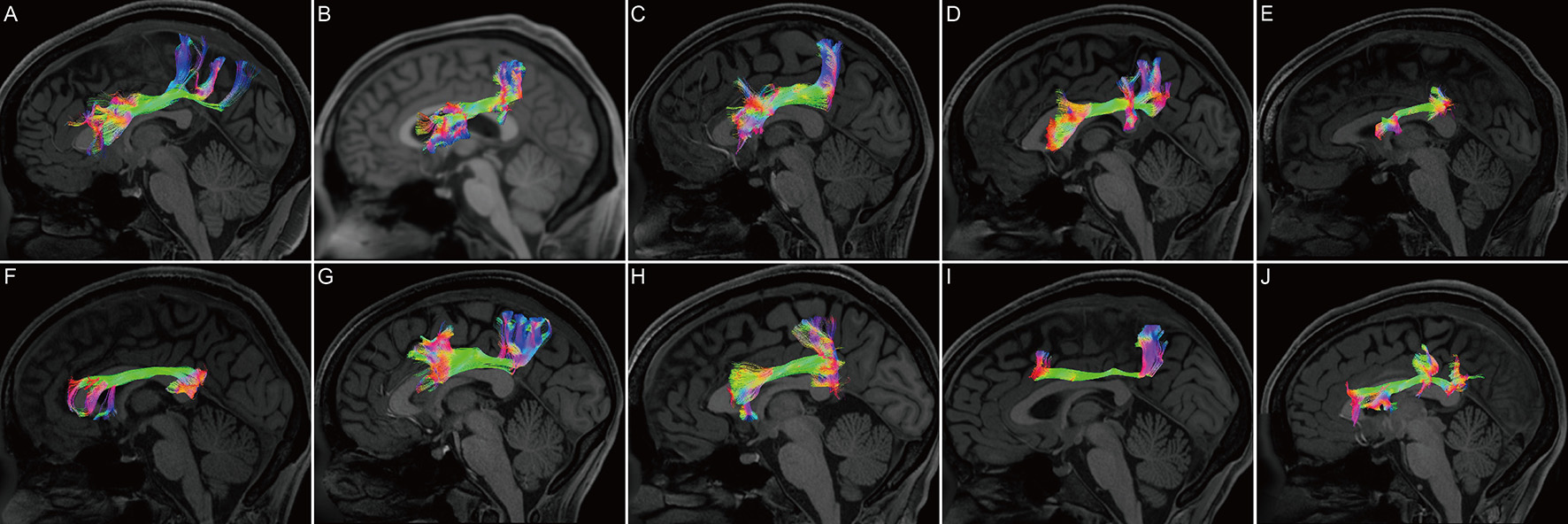

Supplement: Supplementary Figure 4 — In vivo fiber tractography of the IFOF in all 10 subjects. [file Image_4.JPEG]

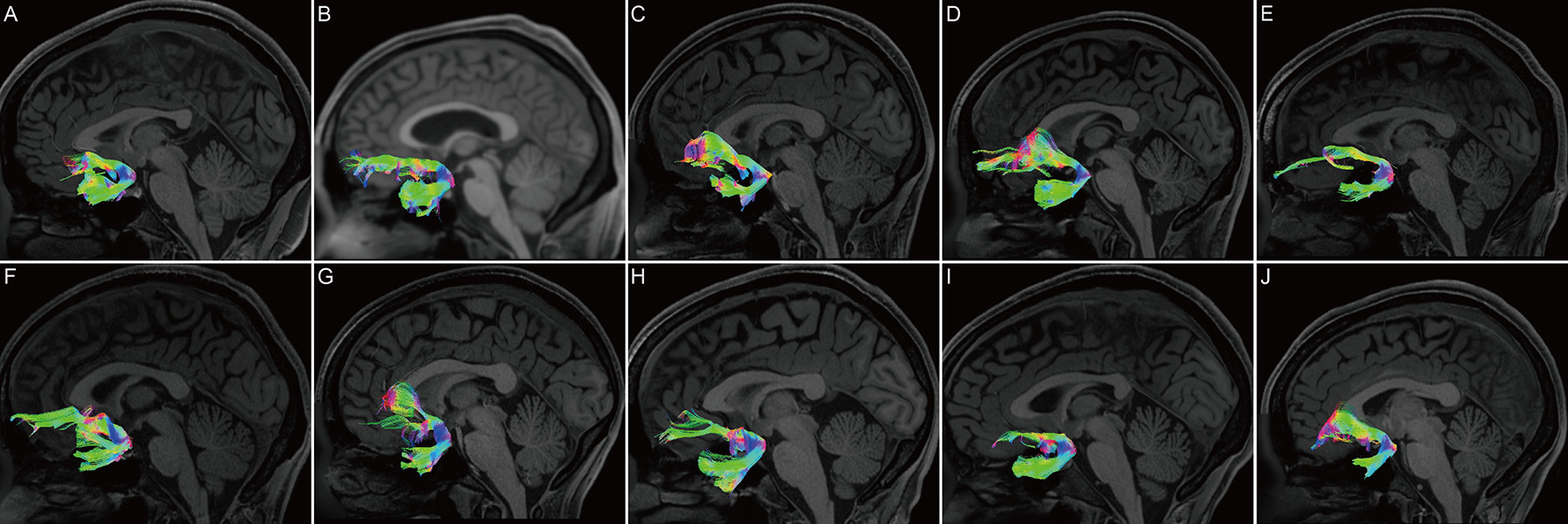

Supplement: Supplementary Figure 5 — In vivo fiber tractography of the SLF-III in all 10 subjects. [file Image_5.JPEG]

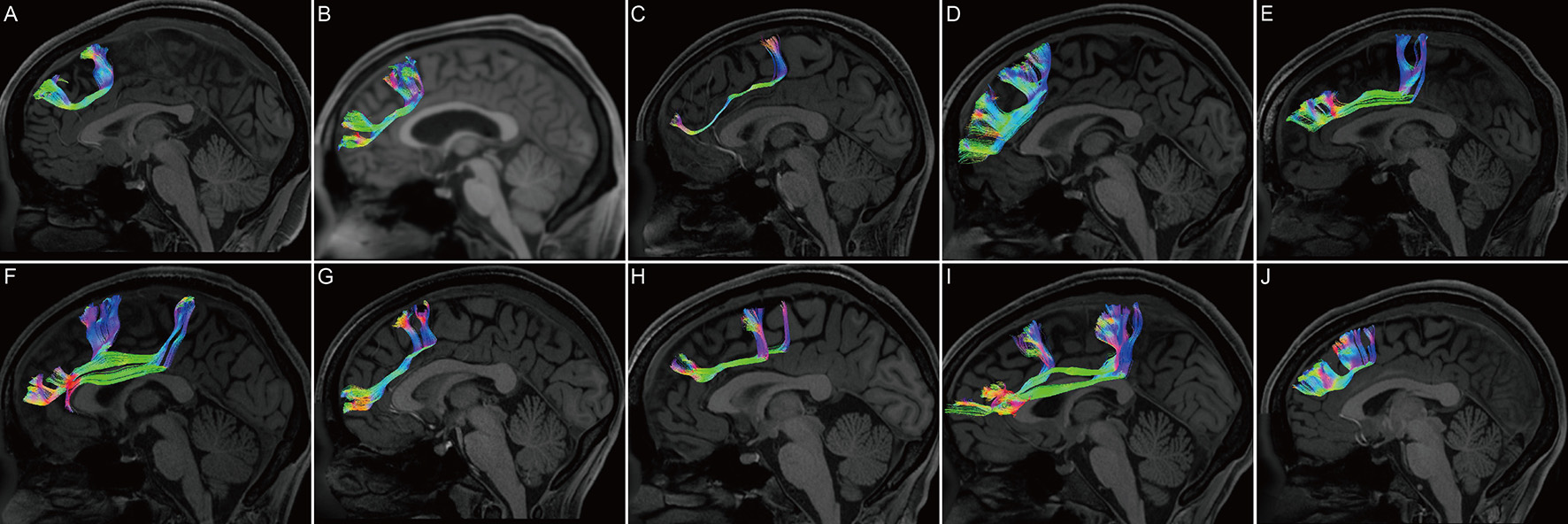

Supplement: Supplementary Figure 6 — In vivo fiber tractography of the UF in all 10 subjects. [file Image_6.JPEG]
